# DNA barcoding: an efficient technology to authenticate plant species of traditional Chinese medicine and recent advances

**DOI:** 10.1186/s13020-022-00655-y

**Published:** 2022-09-28

**Authors:** Shuang Zhu, Qiaozhen Liu, Simin Qiu, Jiangpeng Dai, Xiaoxia Gao

**Affiliations:** 1 Guangdong Province Key Laboratory for Biotechnology Drug Candidates, School of Biosciences and Biopharmaceutics, Guangdong Pharmaceutical University, Guangzhou, 510006 China; 2 School of Pharmacy, Guangdong Pharmaceutical University, Guangzhou, 510006 China

**Keywords:** Traditional Chinese medicine, DNA barcoding, Authentication, Plant species

## Abstract

Traditional Chinese medicine (TCM) plays an important role in the global traditional health systems. However, adulterated and counterfeit TCM is on the rise. DNA barcoding is an effective, rapid, and accurate technique for identifying plant species. In this study, we collected manuscripts on DNA barcoding published in the last decade and summarized the use of this technique in identifying 50 common Chinese herbs listed in the Chinese pharmacopoeia. Based on the dataset of the major seven DNA barcodes of plants in the NCBI database, the strengths and limitations of the barcodes and their derivative barcoding technology, including single-locus barcode, multi-locus barcoding, super-barcoding, meta-barcoding, and mini-barcoding, were illustrated. In addition, the advances in DNA barcoding, particularly identifying plant species for TCM using machine learning technology, are also reviewed. Finally, the selection process of an ideal DNA barcoding technique for accurate identification of a given TCM plant species was also outlined.

## Introduction

Traditional Chinese medicine (TCM), including Chinese herbal medicine, continue to receive international recognition. TCMs have been widely used in the traditional Chinese medical systems and diet therapy. At the same time, TCMs also play an important role in the global traditional health system, not only as food additives, but also as some of the bioactive medical ingredients, such as artemisinin and paclitaxel, etc., which have made a splash in the traditional herbal drug market [1, 2]. In the past decade, the global market for herbal products has expanded, and there has been an increase in the export and import of traditional medicinal products worldwide [3]. Especially, following the outbreak of coronavirus disease 2019 (COVID-19) in 2019, the National Health Commission of the People’s Republic of China recommended a combination of traditional Chinese, such as the Huoxiang Zhengqi capsule [4], Lianhua Qingwen capsule [5], among others, and Western medicine for treating the disease. According to the National Bureau of Statistics of China, the turnover of the Chinese herbal medicine market in 2019 reached 165.3 billion yuan for the domestic market and $6.175 billion for the international side. The increased demand for natural products has created the need to ascertain the authenticity of TCMs’ species.

The authentication of Chinese herbal medicine species began 5000 years ago. For conventional authentication, ancient people generally relied on the flowering and fruiting period of Chinese herbal medicine, as this period is easier and more convenient to authenticate. However, this method faces numerous problems: first, the conventional authentication is limited in species identification without relating to the quality of TCM. Secondly, the required features are only visible during specific periods and need to be authenticated by experts with extensive personal experience. Currently, understanding plant and animal genetics has facilitated the invention of species authentication technologies. DNA barcoding has become an extremely widely used technology in molecular marker-based species authentication technologies, given its standardization, minimization, and scalability. DNA barcoding is now widely used for the rapid identification of TCM species.

In this study, we aim to: ① discuss the dynamics and application prospects of DNA barcoding and its derivative technologies; ② address the issue of accessing the optimal barcodes to authenticate common Chinese herbal species; ③ outline processes for selecting the appropriate technologies for identifying given traditional Chinese herbal species.

## Prevalent adulteration in current Chinese herbs industry

**Fig. 1:**
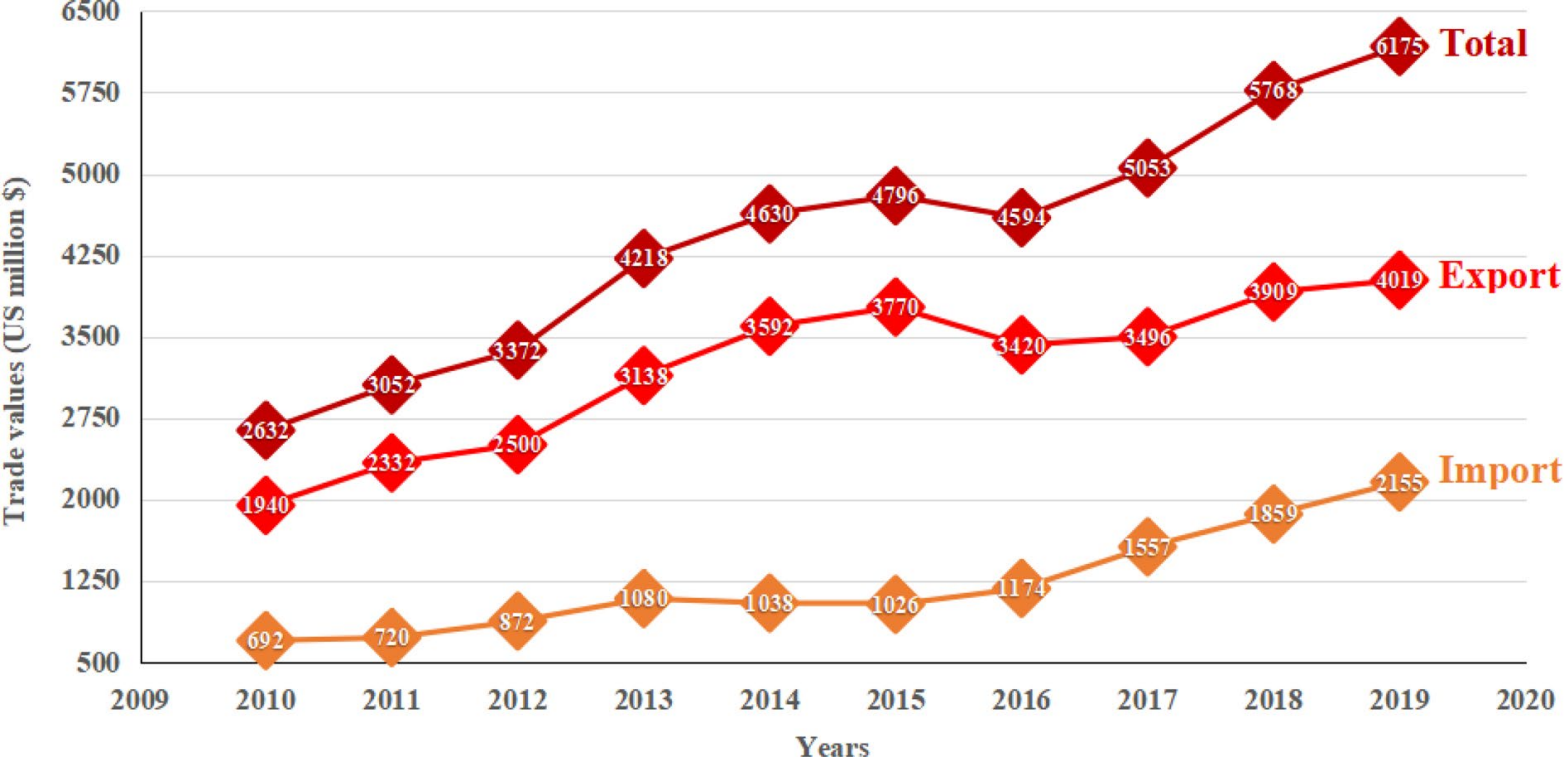
The trends of TCM products trade volume (US million $) in the last 10 years (Data source comes from China Chamber of Commerce for Import & Export of Medicines & Health Products, http://en.cccmhpie.org.cn/)

The TCM industry has rapidly expanded over the past years. Accordingly, the competition resulting from the growing demand for TCM is a key factor of concern. The trend of the TCM industry is shown in Fig. 1. Along with the growing TCM market, there has been an increase in poor quality/fake herbal products, as shown in Table 1. Han et al. investigated 1260 valid samples of 295 medicinal species from 7 TCM markets in China and found that about 4.2% were found to be adulterated [6]. The prevalent problem deteriorated in 2018. Another research investigating 400 seeds for TCM products found that 7.5% of the samples were incorrectly labeled [7].

**Table 1:** Experiment evidence-based existing substitutes or adulterants for Traditional Chinese medical herbs

S.no	Latin name of Traditional Chinese medical herbs	Chinese name	Substitutes or adulterants	References
1	*Lonicerae japonicae Flos*	Jinyinhua	*Eucommiae Folium* or *Lonicerae Flos*	[100]
2	*Angelica sinensis*	Danggui	*Angelica amurensis*	[126]
3	*Pulsatilla chinensis*	Baitouweng	*Potentilla chinensis*	[127]
4	*Ficus hirta*	Wuzhimaotao	*Gelsemium elegans*	[128]
5	*Veronica officinalis* L	Popona	*Veronica chamaedrys* L	[11]
6	*Cynanchi Atrati Radix et Rhizoma*	Cangshu	*Ampelopsis japonica*	[38]
7	*Cynanchi Atrati Radix et Rhizoma*	Baiwei	*Cynanchum mongolicum* or *Cynanchum inamoenum*	
8	*Artemisia annua* L	Huanghuahao	other species of *Artemisia*(e.g*. Artemisia argyi*)	[78]
9	*H. rhamnoides* ssp. *Sinensis*	Shaji	*Nitraria tangutorum*/*Sorbus pohuashanensis* /*Berberis vulgaris*	[107]
10	*Zanthoxylum armatum*	Zhuyehuajiao	*Zanthoxylum schinifolium*	[129]
11	*Panax notoginseng*	Sanqi	*Panax vietnamensis var. fuscidicus*	[20]
12	*Hyoscyami Semen* (seeds of *Hyoscyamus niger* L.)	Tianxianzi	seeds from *Hygrophila salicifolia* (*Vahl*) *Nees*	[130]
13	*Cuscuta australis* R. Br*. and C. chinensis* Lam	Tusizi	*Cuscuta japonica Choisy*	[7]
14	*Atractylodis Rhizoma*	Cangzhu	*Atractylodes koreana* (*Nakai*) *Kitamura*	[62]
15	*Notopterygii Rhizoma et Radix*	Qianghuo	*Sanguisorbae Radix *(*Diyu, Sanguisorba officinalis* L.)	
16	*Tripterygum wilfordii*	Leigongteng	*Celastrus angulatus*	[131]
17	*Arisaematis rhizoma*	Tiannanxing	*Pinellia pedatisecta*	[34]
18	*Tinospora crispa*	Lübaoteng	*Tinospora baenzigeri*	[61]
19	*Rhizoma Paridis*	Chonglou	*Polygonum paleaceum* Wall	[132]
20	*Dipsacus asper*	Chuan-xuduan	*Dipsacus japonicus*	[37]
21	*Akebiae Caulis*	Mutong	*Aristolochiae manshuriensis Caulis*/*Clematis armandi*	[62]
22	*Clematidis Armandii Caulis*	Chuan-mutong	*Akebiae Caulis*	
23	*Alisma orientale*	Zexie	*Alisma plantago-aquatica*	
24	*Bupleuri Radix*	Chaihu	*Bupleurum marginatum*	
25	*Orthosiphon stamineus*	Maoxucao	*Clinacanthus nutans*	[60]
26	*Aquilaria* (*Thymelaeaceae*)	Chenxiang	*Memecylon* sp. (*Melastomataceae*) and *Strychnos* sp. (*Loganiaceae*)	[133]
27	*Berberis aristata*	Xiaobo	*Berberis asiatica*	[134]
28	*Ocimum sanctum*	Shengluole	*Vitex negundo*	[135]
29	*Bacopa monnieri*	Jiamachixian	*Centella asiatica*	
30	*Inulae Flos*	Xuanfuhua	*Inula linariifolia*	[136]
31	*Sophorae Flos*	Huaihua	*Robinia pseudoacacia*	

The emergence of fake and poor-quality TCM has been attributed to the profit-seeking businessmen who gained improper benefits from cheaper and more profitable adulterants with similar shapes or vernacular names that may lead to confusion in species identification, or during the manufacturing process [8–10]. In view of the above, either accidental or intentional, the emergence and increase in the number of fake TCMs on the market are alarming. This problem has an unpredictable impact on the subsequent clinical use and efficacy of Chinese herbal medicine and hinders the progress in the development of precision medicine. Therefore, there is an urgent need for rapid and simple inspection procedures for validating the authenticity of Chinese herbal materials.

## One of the solutions: origins and development of DNA barcoding

The identification of TCM has four major development stages, including sensory evaluation, microscopic identification, physical, and chemical identification (e.g. high-performance liquid chromatography (HPLC) [11]) and DNA-based molecular identification. The former three stages have some limitations in distinguish authentic from fake medicinal materials accurately. The efficacy of the authenticity of TCMs is affected by numerous factors such as the harvesting time, the complexity of the materials, and uncertain bioactive substances. To address this issue, researchers have gradually turned their attention to DNA-based molecular identification of medicinal herbs. DNA barcoding can be used for quality control of Chinese herbal medicine by validating the identity of the corresponding species.

The concept of applying DNA barcodes to identify species was first proposed by Hebert et al. in 2003 [12]. The technique was successfully used in animals and fungi by using the 5ʹ end of the cytochrome oxidase I (COI) from the mitochondrial gene. COI barcode is a haploid, uniparentally-inherited, single-locus gene with high discriminatory power. The gene does not frequently display drastic length variation, strong secondary structure, micro-inversions, or frequent mononucleotide repeats in animals [13]. Combined with well-developed primer sets, the COI barcode method is easy to perform and accurately identifies animal species. However, the COI barcode is unsuitable for plant identification because mitochondrial genes in plants are slowly evolving with very low substitution rates [14].

Researchers have turned their attention to chloroplast and nuclear genomes to find more powerful barcodes in plant species. In the last two decades, major standard single-locus candidate barcodes have been proposed: ITS, ITS2, *mat*K*, rbc*L*, psb*A*-trn*H, and *trn*L*–trn*F, which discriminate plants species with high accuracy. However, it was found that a single barcode was not enough to identify all plants, which necessitated the use of multi-locus DNA barcodes. The Consortium for the Barcoding of Life (CBOL) Plant Working Group proposed a combination of m*at*K and *rbc*L locus to enhance the accuracy of species discrimination [14, 15]. Chen et al. then proposed the ITS2 + *psb*A*-trn*H for the DNA barcoding system for identifying botanical medicinal herbs [16].

The invention of next-generation sequencing (NGS) technology and the emergence of the third-generation sequencing technology have further enhanced the development of the DNA barcode-derived technologies in identifying Chinese herbal medicine species. The current DNA barcode derivative techniques include super-barcoding, meta-barcoding, and mini-barcoding. For example, (i) mini-barcoding can identify species from highly degraded DNA [17, 18]; (ii) meta-barcoding is useful for species richness analysis in a sample containing a mixture of species [19, 20]; (iii) super-barcoding based on plant chloroplast genome is used for species relatedness [21, 22]. The advance in sequencing technology facilitated the improvement of DNA barcoding from detecting a single herb in Chinese medicine to simultaneously detecting several herbs in a Chinese herbal medicine cocktail [23], influencing the selection and utilization of DNA barcoding. These three DNA barcoding-based technologies have broadened the applications and enhanced the practicality of DNA barcoding. Data mining and analysis tools have strengthened the application of DNA barcoding-based technologies, which could effectively identify biological systems in Chinese herbal medicines [24].

Although DNA barcoding technology’s accuracy is increasing daily, this technique faces numerous challenges, such as inadequate standard reference libraries, low success rate of PCR amplification and PCR bias. Despite these problems, the application of DNA barcoding is rising due to its easy operation, high identification success rate and repeatability. For the quality control technology of TCM materials, especially in plant species of TCM, single technology identification of TCM materials and Chinese patent medicines (CPMs) has certain one-sidedness and, thus, combining several technologies is required [23]. Therefore, we recommend combining several identification methods to achieve comprehensive and accurate identification of TCM with DNA barcoding.

## Standard single-locus DNA barcoding

**Fig. 2:**
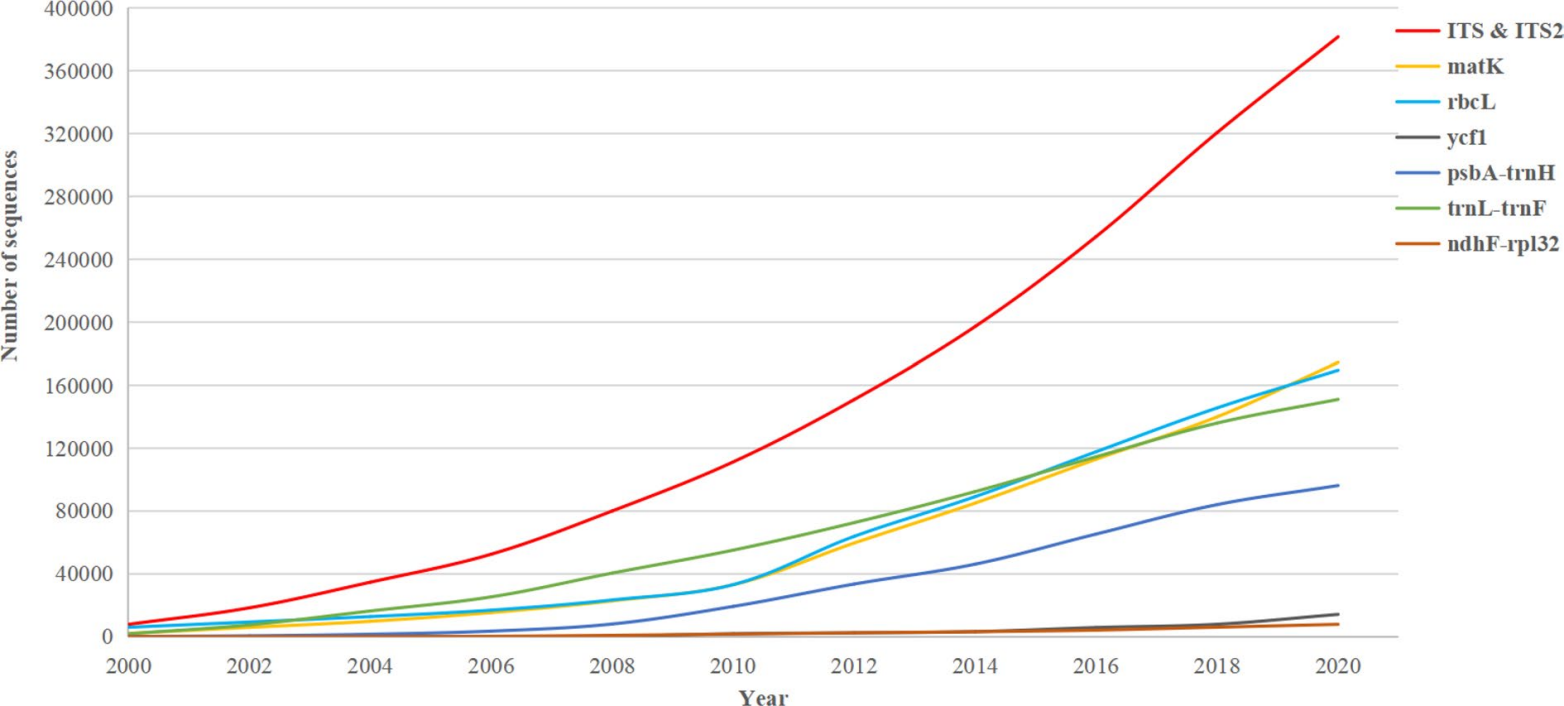
The growth of sequences number of major DNA barcoding of plants in NCBI Genbank

DNA barcoding technology has been used in TCM identification. The number of publications and sequences of different barcodes are rapidly increasing. According to the CBOL Plant Working Group and the number of publications on DNA barcoding between 2010 and 2020, ITS and ITS2, *rbc*L, *mat*K, *trn*L*–trn*F, *psb*A*-trn*H, *ycf*1, and *rpo*C1 are the seven major plant barcodes that have attracted the most attention [25]. Based on the number of DNA barcode sequences in the NCBI database collected (Fig. 2), we found that: (i) ITS and ITS2 are the predominant barcodes. Since 2010, the number of ITS and ITS2 barcodes have been booming. ITS2 region can not only discriminate plant taxa from different plant families but can also distinguish closely related taxa at the genus and species levels [16, 26]. Accordingly, ITS and ITS2 sequences should be utilized more in the future; (ii) *rbc*L (179,816 items), *mat*K (174,431 items), and *trn*L*–trn*F (159,360 items) are the second most dominant barcodes, possibly because they can be used as multi-locus DNA barcodes. The number of publications on *trn*L*–trn*F has increased to 10,000 in 2021; (iii) reasons assumed for the slow growth of *rpo*C1 (15,387 items) and *ycf*1 (16,344 items) might be attributed to the long gene sequences (5709 bp for the *ycf*1 gene of *Nicotiana tabacum*) and lower discriminatory power [27–29]. Several genes, including *atp*F*-atp*H, *ndh*F*-rpl*32, and *psb*K*–psb*I, are potential barcoding candidates. These targets are not so popular in recent publications (less than 1%), probably because of their relatively low discrimination ability, poor universality in different taxa, or unsatisfactory amplification rates [28, 29]. Generally, the sequence number of standard single-locus DNA barcodes is still increasing.

## ITS and ITS2

The internal transcribed spacer (ITS) region of the nuclear ribosomal cistron is the most usually sequenced locus for systematic molecular investigations of TCM at the lower-taxa levels, including the genera, species, and subspecies [30]. ITS offers the advantages of generality, simplification, high copy number, interspecific variability, and intraspecific uniformity [31]. ITS has been used as a universal barcode for distinguishing more than 21,722 plant species and is recommended for validating the authenticity of Chinese herbal medicine [32]. However, certain limitations hinder its application for Chinese herbal medicine barcoding: incomplete concerted evolution as well as difficulties of amplification and sequencing [29]. ITS2, a non-coding nuclear DNA between 5.8S rRNA and 25S rRNA genes, can distinguish closely related taxa at the family, genus, and species levels [26, 33].

ITS2 has strengths in variability, sequence quality and high inter-specific and intra-specific divergence power [16, 26, 34]. ITS2 can identify 92.7% of species correctly in more than 6600 samples obtained from 4800 species in 753 genera [16, 26], such as *Cynanchum auriculatum* [35], *Acanthopanacis* [36], *Dipsacales* [37], *Xueteng* [38]. Besides, the secondary structures of ITS2 provided additional information that enhances the species’ discrimination [39–42]. ITS2 could be used as an alternative mini-barcoding when a full-length ITS is not available and can correctly identify *R. rosea.* [43], and *U. lanosa* [42], among many other species. Currently, effective experimental methods have been developed to avoid fungal contaminants. The Hidden Markov Model (HMM) fungus model proposed by the Florida State University can remove fungal contaminating sequences, enhancing the reliability of the data. Meanwhile, the risk of fungal contamination can be effectively reduced by cleaning the surface of herb roots and scraping off the cortex during sampling. ITS2 could be used to identify herbs in a broader range of plant taxa [26, 44–49], including herbarium specimens with degraded DNA [50]. Accordingly, it is suitable for authentication of traditional Chinese herbal medicine powder.

Although ITS2 has many strengths, it is not ideal for identifying ferns [51, 52]. A major concern is the existence of multiple copies in ITS2 with high levels of within-species and even within-individual sequence differentiation [53]. Furthermore, heterogeneity is an issue for ITS2 due to concerted evolution, which may lead to inaccurate or misleading results [54, 55].

## matK

The high sequence variation and sequencing efficiency rates, evolution, PCR amplification, suitable sequence length, accurate discrimination of angiosperms [53, 56, 57], and the intra and inter-specific divergence distinction in the barcoding gap [58] indicate *mat*K is a useful DNA barcode for plants. This barcode has been used for nearly 5 years to accurately identify *Paeonia suffruticosa* [59], *Veronica officinalis* [11], etc. Despite this, there is a need to develop universal primers for the identification of plant species.

## rbcL

As one of the best potential barcode candidates, *rbc*L can discriminate plants at the family and genus level [60]. The remarkable advantages of *rbc*L are high primer versatility, easy amplification and alignment, and high discrimination power [25]. Recent studies used this barcode to identify plants in *Tinospora* [61], Aceraceae [62], and *Artemisia* [30] genera, among others.

*rbc*L has a relatively low interspecific identification power and is generally used for genetic variation tests. As a separate candidate sequence, it is unsuitable due to this region evolves slowly, implying that its discriminatory power is restricted [33]. Recently, researchers have indicated that poor discrimination of closely related species limits its utility in detecting ingredient substitutions [62], indicating that it should be used alongside other potential barcodes.

## psbA-trnH

The *psb*A*-trn*H barcode, one of the fastest evolving regions in the chloroplast genome, is the interval between both *trn*H (H-GUG) sequence ends and both sides of the *psb*A gene. Usually, *psb*A*-trn*H has better primer universality, a relatively high amplification success rate, and is of good length. Therefore, it can be used to amplify biodegraded samples. These features are especially suitable for the level of species and the higher taxonomic level [63, 64]. *psb*A*-trn*H regions can accurately discriminate members of *Dendrobium* [65] and medicinal pteridophytes (90.2% of species could be accurately identified) [66], and *Mentha haplocalyx* [67].

Meanwhile, due to the repeated loci, pseudogenes, and high insertion/deletion rate, the length of *psb*A*-trn*H vary significantly in different groups [28, 68]. As such, manual correction is required for *psb*A*-trn*H sequence analysis, making it difficult to compare different genera and species.

## trnL–trnF

The *trn*L*–trn*F region is located in the large single-copy region of the chloroplast genome, which consists of the *trn*L gene and the *trn*L*–trn*F intergenic spacer [69]. The *trn*L–*trn*F region has been considered for accurate discrimination of plants at the lower taxonomic levels. The region has a high nucleotide conversion rate, which causes a relatively high genetic variation and provides sites with more systematic taxonomic information. The *trn*L*–trn*F region has been used in systematic taxonomic studies of the *Elytrigia lolioides* [70], the Apocynaceae [71], and *Radix et Rhizoma Rhei* [72], among others. Although mononucleotide repeats can impact sequencing reads in some taxa, this barcode is generally simple to sequence [29].

## Other standard single-locus DNA barcoding

Besides standard single-locus DNA barcoding mentioned above, many other DNA sequences, including *ycf*1 [73], *rpo*C1 [28], *ycf*5 [26], *acc*D [28], *ndh*J [28], and *ndh*F-*rpl*32 [74] have been used for identifying Chinese herbal medicine. This DNA barcoding mentioned above is absent in some major groups of land plants. For instance, *ycf*1 is absent in *Poaceae* [27], whereas *ndh*J is absent in *pines* [75], or it just has lower discriminatory power [25]. Therefore, they are not widely considered accurate plant standard barcodes for identifying Chinese herbal medicine [76].

## Multi-locus DNA barcoding

Several studies have demonstrated the difficulties of discriminating between all plants using a universal DNA barcode [77, 78]. Conflicting results have sometimes been found for related species when using certain barcodes, whereas a single locus barcode does not sufficiently provide the evolutionary distinctions required to distinguish related species. Considering the requirements for accurate discrimination and satisfactory genetic information, multi-locus DNA barcoding is more preferable. Multi-locus DNA barcoding is gradually being accepted for accurate identification of TCM.

Multi-locus DNA barcoding represents a practical solution to reach a trade-off between universality, sequence quality, discrimination, and cost. At first, Kress et al*.* suggested that ITS + *psb*A-*trn*H have the potential to discriminate against numerous plant species [33]. The CBOL Plant Working Group evaluated seven chloroplast genomic regions and proposed the 2-locus *mat*K + *rbc*L plant barcode in an international conference since *mat*K provides high resolution but less universality, whereas *rbc*L provides high universality but less species resolution [25]. Researchers believed combining these two barcodes could achieve maximum species discrimination [29]. To achieve higher discrimination in closely related species, the China Plant BOL Group proposed to add the nuclear ITS (internal transcribed spacer) to the *mat*K + *rbc*L combination [79]. Chen et al. first proposed the ITS2 sequence as a universal barcode for medicinal plant identification and the ITS2 + psbA-trnH combination as a DNA barcoding system for identifying botanical medicinal herbs [26]. The advantages of multi-locus barcoding are that the results can be mutually verified and complemented and can discriminate among numerous species. This combination demonstrated the excellent reliability for species authentication, and researchers have identified more than 23,262 different species for Chinese, Japanese, Korean, and European herbal medicine [36, 79]. Among the top ten Chinese herbal medicine and decoction of processed materials exported in 2019, five were identified using multi-locus barcoding: *Pinellia hunanensis* using *mat*K + *rbc*L [29], *Panax ginseng* C.A*. Meyer* and *Radix Astragali* using *psb*A*-trn*H + ITS [26, 81], *Zizyphus jujube* using ITS2 + *psb*A-*trn*H [82], *Angelica sinensis* using ITS + *rbc*L + *mat*K + *psb*A-*trn*H (slightly better discriminatory power than ITS) [27].

Although it still failed to meet the original goal of the universality of DNA barcoding and the differentiation of closely related complex groups is still uncertain, the multi-locus approach of combining different barcodes has been successful in certain cases, including species discrimination [28, 29]. In general, the discrimination of Chinese herbal medicine species using DNA barcoding is still under research and development.

## Super-barcoding

In 2008 at the Botany without Borders conference, it was pointed out that the chloroplast genome contains about as much information as the short mitochondrial barcode sequence used in animals [83]. With the need for accurate identification of certain closely related species, scholars proposed the concept of super-barcoding (ultra-barcoding), which means sequencing the whole plastid genomes as a barcode [83]. Here, the whole organelle’s genome or large (greater than 5 kb) contiguous portions of the nuclear genome are sequenced and assembled [21]. Compared with the nuclear genome, the chloroplast genome is smaller and has a higher interspecific and lower intraspecific divergence [53]. Therefore, sequencing the chloroplast genome is more common.

Super-barcoding is a promising approach for identifying Chinese herbal medicine and has many advantages, including ① circumventing gene deletion problems, locus choice, and low PCR recovery rate often encountered in the conventional barcoding [84], ② higher resolution, and better versatility [21], and ③ can be supplemented the traditional DNA barcoding. Compared with traditional barcoding, super-barcoding enhances the identification of closely related groups, including accurate discrimination of subspecies. For instance, the super-barcoding was shown to successfully distinguish closely related species such as *Araucaria* spp. (Aruacariaceae) [85] and *Echinacea* (Asteraceae) [86], especially for taxonomically complex groups, e.g., *Camellia* spp. (Theaceae) [87], Chinese herbal medicine *Epimedium* spp. (Berberidaceae) [88], *Fritillaria* spp. (Liliacae) [89] and *Taxus* (Taxaceae) [84]. Super-barcoding often uses high-throughput next-generation sequencing (generally in massively parallel sequencing) to scan the genome and generate a reliable sequence of high copy number regions. It gets more information sites and expands the traditional barcode regions (standard single-locus barcoding) to their full, many-kilobase length [21]. This method increases the density and phylogenetic coverage of the complete plastid genome sequence and is expected to accurately identify traditional Chinese herbal medicines.

The main stumbling blocks for super-barcoding are the cost and the requirement for high quality and quantity of DNA, large next-generation sequence data generated as well as large amounts of next-generation sequence data needed to deal with [21]. Besides, the variation present over short regions may be too low to distinguish recently diverged taxa because evolution is generally slow in the plastid genome [90].

**Fig. 3:**
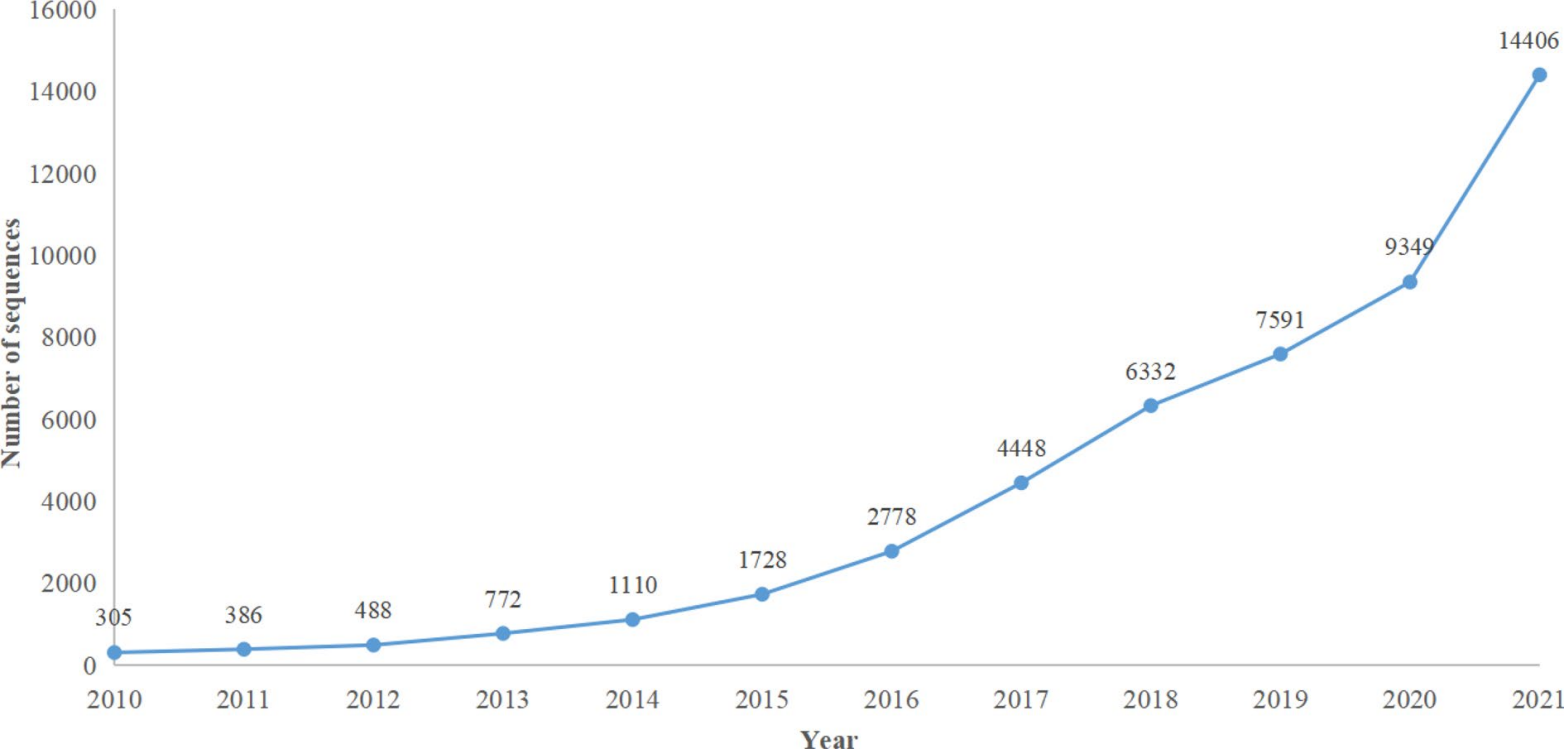
The total number of complete plant chloroplast genome sequences to GenBank from 2010 to 2021

With the increasing number of the whole chloroplast genomes in GenBank (Fig. 3), it is foreseeable that the super-barcoding application in TCM herbs will be wider than standard plant DNA barcoding in the coming years. Super-barcoding does not override the need for continued use of traditional barcode methods but rather provides necessary data to examine variation below the species level [21]. Continued advances in sequencing technology may make super-barcoding the choice for plant identification at the intra-species or population levels in the future [32].

## Meta-barcoding

Currently, a new DNA barcoding-based method for rapidly and simultaneously identifying numerous taxa (i.e., different Chinese medical herbs) in a single environmental sample (i.e., multi-ingredient traditional CPMs) has been developed. The emergence of DNA meta-barcoding has been facilitated by the availability of the next-generation sequencing platforms and the need for high-throughput taxon identification. In 2012, meta-barcoding was defined as “designate high-throughput multispecies (or higher-level taxon) identification using total but degraded DNA extracted from an environmental sample (i.e., soil, water, feces, etc.)” [91]. DNA meta-barcoding to identify samples include ① collecting mixed-species environmental DNA samples (obtain raw materials), ② sample processing (DNA extraction and PCR amplified sequences), ③ next-generation sequencing, ④ data analysis (obtain clean data and OTUs from raw data), and ⑤ species identification [92].

The greatest advantage of DNA meta-barcoding is its ability to identify every species in a complex sample or processed mixtures simultaneously. Even so, the application of DNA barcoding and conventional analytical methods are considerably limited [23]. The CPMs’ components are complex, and the sample DNA is degraded seriously. Thanks to high accuracy, DNA meta-barcoding can measure the components of CPMs simultaneously with high coverage and, thus, override the aforementioned problems. Thus, meta-barcoding is increasingly used for detecting CPMs’ components. For instance, an Australian team identified barcodes for CPMs, including animal and plant medicines, in the form of tablets, capsules, powders, and herbal teas [93]. The potential power of DNA meta-barcoding is the ability to reveal plant species diversity within processed products. For example, it has successfully identified *Veronica* species, and detected substitution or admixture of other *Veronica* species in *V. officinalis* herbal products [11]. The main medicinal plants in the CPMs, including *Lonicera japonica* Thunb*.*, *Forsythia suspensa,* and *Angelica pubescens* have been identified using DNA meta-barcoding [94].

However, the potential applications of DNA meta-barcoding are limited by the PCR success rate and the considerable investment in building comprehensive taxonomic reference libraries [95]. Also, sequencing errors in high-throughput sequencing are still inevitable.

DNA meta-barcoding can simultaneously detect multiple species from complex samples and facilitates species diversity assessment in processed products, which is extremely important for validating the authenticity of products in Chinese medicinal plants [23]. Therefore, this method can rapidly and accurately identify TCM, including Chinese herbal medicine. However, meta-barcoding should be used in combination with other appropriate chemical methods.

## Mini-barcoding

Due to the common DNA degradation in TCM, it is difficult to obtain the full-length sequence data using the traditional standard barcodes. Mini-DNA barcoding technology can override this limitation. Mini-barcoding can utilize incomplete, relatively short sequences from standard DNA barcodes to identify different species, which is useful for degraded DNA preservation. Overall, it improves the identification accuracy of species [96, 97]. One of the most common mini-barcode regions is *trn*L (UAA) intron. The P6 loop of the chloroplast *trn*L (UAA) intron can be robustly amplified with highly conserved primers from degraded DNA samples [95, 98]. Therefore, it can be used to identify the components in processed medicinal materials up to the species or genus level [18]. Other common mini-barcoding regions include the shorter *ycf1*a and *ycf1*b [99], short region in ITS2 [100–102], and short region in *rbc*L [103].

Mini-barcoding has been successfully used to identify traditional Chinese herbal ingredients such as *Angelicae sinensis radix*, *Ligusticum sinense*, and *Notopterygium incisum,* among others [102]. Currently, it has been applied to identify the traditional medicinal plant *Rhodiola* (Crassulaceae) [43], distinguish members of the *Apiaceae* family [104], and discovery of numerous species in *Metazoa* [105] and more natural herbal products [17]. Nonetheless, the few nucleotides often limit taxonomic discrimination using mini-barcoding, resulting in the main limitation of mini-barcoding being the resolution [97, 106]. An acceptable resolution not only depends on the accurate species identification but also on whether reference sequence data is sufficient. To fully maximize the power of mini-barcoding, more reference sequences need to be added to the databases.

Due to the shorter molecular markers of mini-barcode, different physicochemical technologies can be combined to identify Chinese herbal samples rapidly. For example, sea buckthorn (*Hippophae*) were accurately identified in Chinese herbal products using a combination of mini-barcoding and high-resolution dissolution (HRM) [107]. In the future, Mini-barcoding may become a complementary barcoding technique to identify traditional Chinese herbal medicine [18].

## Applications of the current DNA barcoding techniques for authenticating Chinese herbal medicine

We selected 50 common Chinese herbal medicines in the Chinese pharmacopoeia based on the published papers on TCM and DNA barcode identification in recent years. The barcode choices are shown in Table 2. We found that DNA barcoding has been used for large-scale identification of Chinese herbal medicines. We also summarized the preferred barcodes for different families or genera based on published papers (Table 3). Each species has a specific most ideal barcode, called “specific barcode”. A specific barcode may include one of the single-locus barcodes (e.g., *mat*K or *psb*A*-trn*H) or could be based on new markers never used before [52]. Tables 2 and 3 summarize the recent developments in DNA barcoding for identifying Chinese herbal medicine species and the preferred DNA barcode for specific plants.

**Table 2:** Applications of DNA barcoding and DNA sequence-based markers in the identification of Chinese herbs

Name	Plant parts	Chinese herbs name (Pinyin)	Barcoding markers used	References
*Ephedra*	–	mahuang	ITS2	[80]
*Salvia miltiorrhiza*	Root and rhizome	danshen	ITS2	[51]
*Angelica sinensis*	Root	danggui	ITS2 and *psb*A*-trn*H	[102]
*Bupleurum*	Root	chaihu	ITS2	[137]
*Eucommia*	Bark	duzhong	ITS2	[138]
*Notoginseng*	Root	sanqi	ITS2	[139]
*Szechwan lovage*	Rhizome	chuanxiong	ITS2 and *psb*A*-trn*H	[140]
*Schisandra*	Fruit	wuweizi (beiwuweizi)	ITS2	[141]
*Atractylodes*	Rhizome	baizhu	ITS2	[142]
*Astragalus mongholicus*	Root	huangqi	ITS2	[143]
*Baical skullcap*	Root	huangqin	*psb*A-*trn*H	[144]
*Angelica dahurica*	Root	baizhi	ITS	[145]
*Isatis*	Root	banlangen	ITS2	[146]
*Peony* root, white	–	baishao	ITS2	[59]
*Peony* root, red	–	chishao	ITS2	[59]
*Carthamus tinctoriu*s L.	Flower	honghua	ITS2	[147]
*Coptis chinensis* Franch	Rhizome	huanglian	ITS2	[26]
*Cape jasmine*	Fruit	zhizi	*mat*K	[120]
*Codonopsis*	Root	dangshen	ITS + *mat*K	[32]
*Magnolia oficinalis*	Bark	houpo	ITS2	[138]
*Uncaria*	Stem	gouteng	ITS and ITS2	[42]
*Platycodon*	Root	Jiegeng	ITS2	[148]
*Angelica pubescens*	Root	duhuo	ITS	[32]
*Thomson kudzuvine*	Root	fenge	ITS2	[26]
*Kudzuvine*	Root	gegen(yege)	ITS2	[26]
*Gastrodia*	Rhizome	tianma	*matK*	[120]
*Evodia*	Fruit	wuzhuyu	ITS2	[47]
*Fleeceflower*	Root	heshouwu	*mat*K + *rbc*L + *psb*A*-trn*H + ITS2 or *psb*A*-trn*H	[32]
*Coix*	Seed	yiyiren	ITS2	[7]
*Andrographis*	–	chuanxinlian	*mat*K	[120]
*Lightyellow sophora*	Root	kushen	ITS2	[147]
*Achyranthes bidendata*	Root	niuxi	ITS2	[26]
*Anemarrhena asphodeloides*	Rhizome	zhimu	*trn*L*-trn*F	[32]
*Akebia*	Stem	mutong	ITS2	[42]
*Aucklandia*	Root	muxiang	ITS2	[26]
*Atractylodes lancea*	Rhizome	cangzhu	ITS2	[42]
*Lycium barbarum* L.	Fruit	gouqizi	ITS	[42]
*Corydalis*	Rhizome	yanhusuo	ITS2	[26]
*Typhae*	Pollen	puhuang	ITS2	[147]
*Polygonum cuspidatum*	Rhizome and root	huzhang	*psb*A*-trn*H	[32]
*Moutan*	Bark	mudanpi	ITS2	[1]
*Drynaria*	Rhizome	gusuibu	*psb*A*-trn*H	[32]
*Prunella vulgaris* L.	Rhizome and root	xiakucao	ITS2	[51]
*Amomum*	Fruit	sharen	ITS2	[147]
*Belamcanda chinensis*	Rhizome	shegan	*rbc*L	[149]
*Piper longum* L.	–	bibo	*mat*K + ITS	[149]
*Sophora*	Flower	huaihua	ITS2	[33]
*Piper Nigrum* L.	–	hujiao	*mat*K + ITS	[150]
*Zanthoxylum bungeanum*	Pericarp	huajiao	ITS2	[150]
*Clematis armandii*	Stem	chuanmutong	ITS2	[52]

**Table 3:** Preferred loci for family or genera level in plants identification

	Family	References	Family	References	Genus	References	Genus	References
ITS and ITS2	Verbena officinalis	[151]	Caprifoliaceae	[152]	*Begoniaceae*	[153]	*Crassulaceae*	[141]
	Rubiaceae	[154]	Apiaceae	[140]	*Paris*	[155]	*Ilex*	[156]
	Rutaceae	[157]	Euphorbiaceae	[49]	*Amomi Fructus*	[158]	*Pulsatilla*	[127]
	Rosaceae	[49]	Asteraceae	[46]	*Viburnum*	[159]	*Panax*	[81]
	Malvaceae	[160]	Aristolochia	[161]	*Astragalus*	[162]		
	Zingiberaceae	[163]	Gentianaceae	[164]				
*mat*K	Fabaceae	[119]	Juglandaceae	[165]	*Gardenia*	[125]	*Ardisia*	[58]
	Araceae	[166]						
*psb*A*-trn*H	Polygonaceae	[167]	Lauraceae	[168]	*Rhododendron*	[169]	*Aconitum*	[170]
					*Cistanche*	[171]	*Dendrobium*	[65]
*ycf*1	Cymbidium	[27]						
super-barcoding	Asteraceae	[53]	Schisandra chinensis	[172]	*Aconitum*	[173]	*Dendrobium officinale*	[174]
	Cymbidium	[87]						
*psb*K*-psb*I					*Dendrobium*	[175]		

In recent years, with the continuous development of high-throughput sequencing technology and DNA barcode research, genomics is increasingly being applied to identify Chinese herbal medicine. Genome capture of nuclear markers has attracted researchers’ attention, and the genome skimming approach can bridge the gap between the standard barcode and genome sequencing [108]. Research on TCM genomics with TCM original species has achieved tremendous success [109–111]. However, the huge workload posed by data processing and sequencing cost is significantly higher than the cost of common barcode sequencing. It is not necessary to use genomics to identify plant species of TCM.

Regarding data mining, some studies suggest that machine learning methods can identify species using DNA multi-locus barcoding or just standard single-locus barcoding [112, 113]. Machine learning is based on building algorithms that receive input data for calibration and statistical analysis of the output value within an acceptable range. The common DNA barcode analysis methods in machine learning include BLOG (Barcoding with LOGic) and WEKA. Currently, eight *Dalbergia* timber species use SMO, a classifier, as part of the WEKA approach [114–116]. This approach resulted in the best (98–100%) discrimination, and the two-locus combination of ITS2 + *psb*A-*trn*H showed the highest success rate [112]. The character-based DNA barcode method in BLOG 2.0 was applied to classify members of the *Epimedium* genus. It was found that *psb*A*-trn*H + ITS and *psb*A-*trn*H + ITS + *rbc*L exhibited the highest identification ability [117]. Machine learning and DNA barcoding technology are intertwined in two different fields. With the help of machine learning, the application of DNA barcoding technology in the identification of TCM will be strongly promoted in the future.

The increasing use of DNA barcoding is due to the emergence of more available sequence data and information for machine learning and the regular update of public DNA barcode databases. Currently, DNA barcoding is widely used in authenticating medicinal materials in TCM, inseparable from the continued development of public barcode databases. As one of the most common databases, Chen et al. constructed a large-scale DNA barcode platform (http://www.tcmbarcoding.cn), widely used to identify herbal materials for varied needs [79]. This database is a collection of barcode sequences for herbs, including Chinese, Japanese, Korean, Indian, and European pharmacopeia species [7, 62, 80, 118, 119]. This reliable system for DNA barcoding of herbal materials has been established based on a two-locus combination of ITS2 + *psb*A-*trn*H loci barcode and contains 78,847 sequences for 23,262 species. To be specific, this platform has been used in TCM enterprises for raw herbal material identification [32]. This greatly speeds up the industrial procurement of raw materials and provides a standardized method for industrial identification of Chinese herbal medicine. That aside, a library of genuine Lingnan medical herbs DNA barcodes based on ITS2 has been constructed, containing 1276 sequences from 309 species from southern China [51]. It is used to identify genuine Lingnan medical herbs and the authenticity of the constituent ingredients, improving the standard of the Chinese medicine market. The Chinese University of Hong Kong built a Medicinal Materials DNA Barcode Database (MMDBD, http://www.cuhk.edu.hk/icm/mmdbd.htm), encompassing other barcodes such as *rbc*L in seed plant species [120], ITS2 + *psb*A*-trn*H [32, 43], and *rbc*L + *psb*A*-trn*H [60, 120]. All these public DNA barcode databases provide a platform for identifying TCM plant species. It is vital to update and maintain a public, standard DNA barcode database. Besides, good practice protocols are needed to ensure such databases provide clear information in this respect [32].

The development of new apparatuses in recent years has also made this technology more practical. Based on the need to automate the identification of TCM, digitize and integrate the identification of herb-based species, the new Chinese herbal DNA barcoding high-throughput gene sequencing machine (HMBI-G30) was developed successfully. This new apparatus can test up to 30,000 samples in a single run with high accuracy and reliability, facilitating one-stop sequence processing. Meanwhile, high-curvature nanostructuring-based electrochemical herb sensor (nanoE-herb sensor) is a direct, sequencing-free method for identifying herbal species accurately [121]. The use of such portable and cheap sensors facilitates rapid identification of other plant species in herbal medicines. NanoE-herb sensor has been for the ITS2 sequence to accurately identify herbal *C. sativus* in a mixture of counterfeit products. The continuous innovation of new instruments based on the DNA barcode principle has facilitated the identification and standardization of Chinese herbal medicine.

**Fig. 4:**
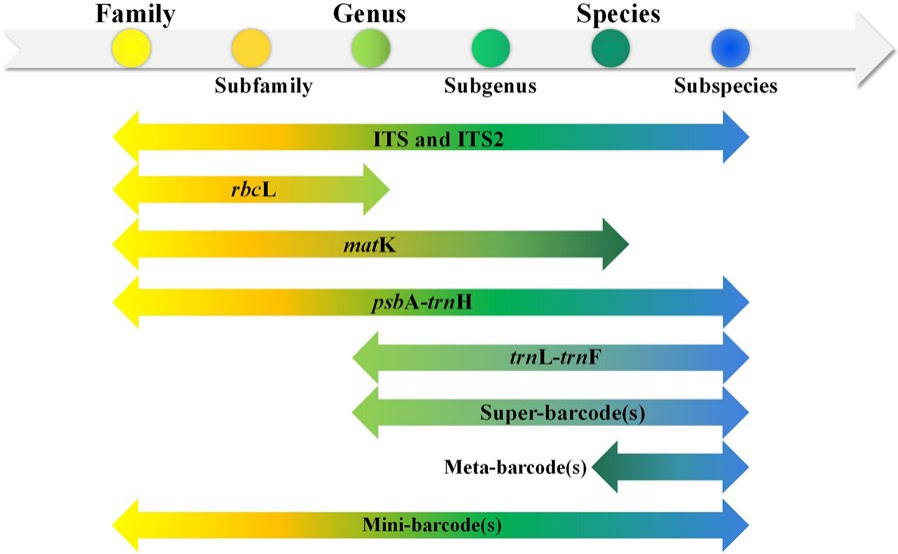
Common DNA barcoding are typically used to identify different ranges of taxonomic units

**Fig. 5:**
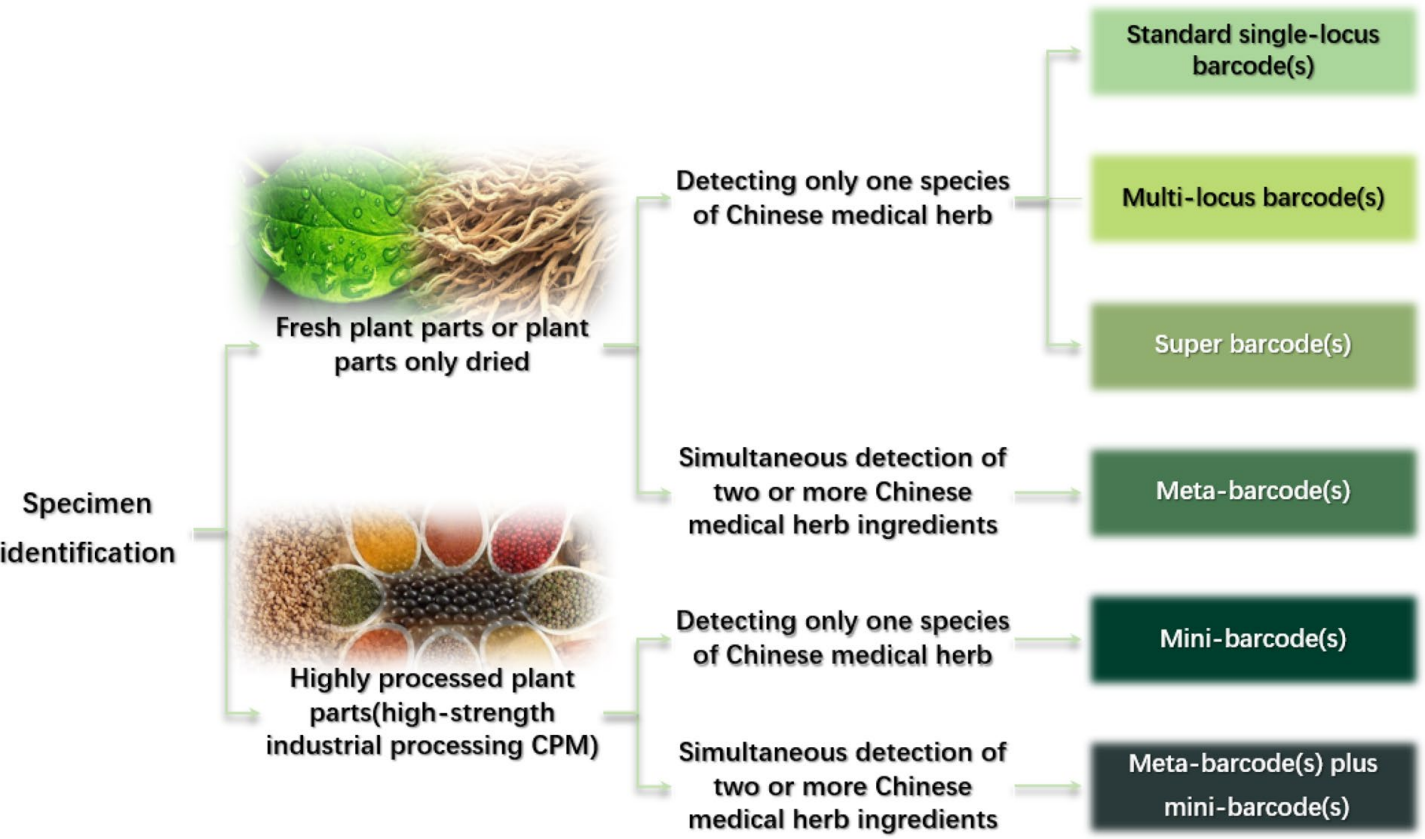
A schematic diagram about how to choose the appropriate DNA barcoding technology for the Chinese herbal medicine identification

Therefore, we hold the following views regarding the application prospect of DNA barcoding and its derived technologies. From specific species to families and genera, our conclusion is captured in Figs. 4 and 5. Overall, a common DNA barcode can be used for organisms at different taxonomic units (Fig. 4). Since the standard single-locus barcodes ycf1 and rpoC1 are ambiguous as described in the papers, they are generally used in combination with other barcodes in the multi-locus barcoding approach. Given that they are not used alone, they are not listed in Fig. 4. It only shows the ranges of common applications but does not exclude the possibility that some barcodes have higher or lower accuracy in identifying certain species or members of a given genus. For the relatively new barcoding technologies, the super-barcoding and meta-barcoding have high accuracy and resolution in the identification of species at lower taxonomic levels. Super-barcoding and meta-barcoding are rarely used for primary screening but for verification or validation of doubtful results generated by the conventional standard single-locus barcoding or multi-locus barcoding techniques. The mini-barcoding has gained wider recognition because overly degraded DNA is difficult to identify using conventional single-locus barcoding or multi-locus barcoding techniques. Therefore, mini-barcoding is directly used for identifying plant taxonomic groups and, literally, the classification range is wider.

Based on the ranges of application of DNA barcoding shown in Fig. 4 and the characteristics of each derivative technology summarized above, we provide a schematic procedure for selecting the ideal DNA barcode for identifying Chinese herbal medicine (Fig. 5). In this diagram, the high processing includes but is not limited to injections, pills, tablets, granules, powders, plasters, capsules, and other dosage forms. Traditional DNA barcoding is preferred to identify TCM herbs. It is recommended to use the traditional standard simple-locus barcoding in a single sample. If this method fails and accuracy is needed, then super-barcoding should be applied. Meta-barcoding is the technique of choice for the simultaneous identification of multicomponent samples. Overall, meta-barcoding and super-barcoding have become more and more common for identifying species in Chinese herbal medicine [22]. Research on mini-barcoding has broadened the application of DNA barcodes and has broadened the prospects for identifying Chinese herbal medicine materials from highly degraded DNA [99]. However, it is hard to identify all components in Chinese medicinal materials simultaneously using only the mini-barcoding [17]. Despite this, a combination of meta-barcoding and mini-barcoding has become a new trend for identifying proprietary Chinese herbal medicine, which has greatly promoted analyzing the composition of CPMs.

## Future perspectives in DNA barcoding for validating the authenticity of TCM

DNA barcoding and its derivative technologies in combination with other technologies (e.g., machine learning, electrochemical sensors, etc.) have achieved tremendous results in identifying Chinese herbal species. In the present paper, we summarized the development of DNA barcoding, both single and multi-locus barcoding widely used in validating plant species in TCM. Our research mainly focused on the potential development and application of DNA barcoding derivative technologies, including super-barcoding, meta-barcoding, and mini-barcoding. By carefully analyzing the application of the DNA barcoding derivative technologies, we developed a schematic procedure for selecting the ideal DNA barcoding technique for identifying given species in TCM. The DNA barcoding prospects and its derivative technologies were also suggested.

As sequencing technologies evolve, sequencing costs and error rates decrease, whereas the coverage and sensitivity in sequencing increase. Also, the speed of sequencing increases while the quality of data increases. However, it must be acknowledged that, given the complexity of the preparation of Chinese herbal medicines, DNA barcoding is not a panacea for validating the authenticity of TCM. Looking ahead, the following issues need to be refined to advance the development of DNA barcoding technologies: ① Sampling and classification: the sampling protocols for DNA barcoding should be standardized. For example, the concept of Daodi medicinal materials has been compared to the “terroir” concept, which means that the specific herbs came from designated geographic regions where conditions including climate, soil, and technologies of cultivation in the case of plants [122, 123]. How can medicinal Daodi materials and non-medicinal Daodi counterparts be differentiated despite being sourced from the same species? ② With the development of NGS and its wide use, the DNA barcoding developments of Chinese medicinal materials are gravitating towards genomics, which will contribute to the development of herb genomics [124]. Can these DNA barcode-based technologies potentially upgrade from authenticity validation or detection of adulteration to authentication of herbal medicines’ quality based on epigenomics or epigenetics information? If molecular information like DNA methylation or histone modifications could help authenticate quality, it will widen the application of these DNA barcode-based technologies, which are essential for developing TCM precision medicine. In general, we advocate for the following: ① maintaining and updating the global plant DNA barcode library; ② updating the standardizing protocols for sampling and classifying process and ③ assessing the feasibility of combining genomics and biological technologies such as transcriptomics (specific expression subset analysis) and proteomics (specific proteome) [125]).

Recent reports and scientific studies have highlighted the widespread adulteration and substitution of ingredients in TCM, which threatens the safety of consumers. In this review, we summarized the strengths and limitations of each DNA barcoding technique and its derivative identification technologies as well as recent developments in sequencing technology, data mining, databases, and new tools related to DNA barcoding. The systematic process for selecting the appropriate barcode or derivative technologies analyzing TCM was also outlined. As a fast and effective method of identifying Chinese herbal medicines, DNA barcoding and its derivative technologies can be combined with several other methods. In the near future, these technologies will be used for quality control of TCM at the species level, which promotes the development of precision of TCM and speeds up the standardization and identification of herbal medicine.

## Data Availability

Not applicable.
